# Comparative analysis of hydrophobicity and dentin adhesion ability in *Candida albicans* strains

**DOI:** 10.1590/1678-7757-2024-0154

**Published:** 2024-09-09

**Authors:** Tatiana Teixeira de MIRANDA, Leonardo RODRIGUES, Carlos Augusto ROSA, Ary CORRÊA

**Affiliations:** 1 Universidade Federal de Alfenas Alfenas Minas Gerais Brasil Universidade Federal de Alfenas, Alfenas, Minas Gerais, Brasil.; 2 Universidade Federal de Minas Gerais Belo Horizonte Minas Gerais Brasil Universidade Federal de Minas Gerais, Belo Horizonte, Minas Gerais, Brasil.; 3 CTNANO UFMG Belo Horizonte Minas Gerais Brasil CTNANO UFMG, Belo Horizonte, Minas Gerais, Brasil.

**Keywords:** Dentin adhesion, Hydrophobicity, Candida albicans

## Abstract

Adhesion to dentin is a first step for a successful microbial root canal colonization. Cell hydrophobicity seems to have some influence in the *Candida species* adhesion to surfaces.

**Objective:**

to measure cell surface hydrophobicity and to investigate the adherence ability to human dentin among *Candida albicans* strains isolated from root canal and lingual dorsum via an *in vitro* study.

**Methodology:**

adhesion was quantified in function of dentin area covered by blastospores and/or hyphae presence detected by epifluorescence microscope. Cell surface hydrophobicity was estimated by assessing the percentage migration of cells from an aqueous phase to a hydrocarbon phase. Contact angles were measured by the sessile drop technique on the dentin surface using a contact angle measurements apparatus. We also examined the correlation between adhesion ability and hydrophobicity.

**Results:**

although there was some intra-species variation in cell surface hydrophobicity, most isolates were characterized by moderate hydrophobicity. There was no significant difference in this parameter when the isolation niche was considered. Both root canal and lingual dorsum yeasts were able to adhere to dentin. No association was found between the strains’ site of isolation and adhesion. Moreover, cell surface hydrophobicity and adhesion ability were not correlated.

**Conclusion:**

although hydrophobicity can influence *Candida albicans* virulence in many ways, this study suggests that this parameter by itself was not a good predictor of adhesion to dentin.

## Introduction

Endodontic diseases are mainly of microbial origin. Instead of being driven by a single bacterial or fungal species, the disease progresses through an orchestrated collaboration of a community of microorganisms, all of them commonly present in the commensal oral microbiome.^1-4^

*Candida albicans* and, to a lesser extent, other opportunistic yeast species are commonly found in the oral cavities of both adults and children, with the reported prevalence ranging from 15 to 75%.^5-7^ These microorganisms can be isolated from different oral sites, including the tongue, cheeks, palatal mucosa, caries, restorative materials, dentures, periodontal tissues, and even root canals.^8-12^

Although previous studies^11,13^reported a prevalence of *C. albicans* in endodontic infections ranging from 0.5% to 55%, the involvement of yeast species in periapical disease remains uncertain. The versatility of these yeasts in adapting to diverse environmental conditions, adhering to surfaces such as dentin and root filling materials, secreting hydrolytic enzymes, undergoing morphological transitions, and forming biofilms, as well as their ability to evade host defenses, suggests that they can act as opportunistic endodontic pathogens.^14^

The relative cell surface hydrophobicity (CSH) of *C. albicans* and other opportunistic yeast species is closely associated with the initial colonization of host surfaces and plays a significant complementary role in regulating these early events.^15^ Previous studies^16-18^ have investigated whether a correlation exists between CSH and the adhesion of *Candida* spp. to buccal epithelial cells and inert polymeric surfaces such as denture prostheses.

The relationship between CSH and adhesion has been extensively examined across different substrates and among different *C. albicans* strains.^19-21^ However, there is a notable lack of information regarding the hydrophobicity profile of *C. albicans* isolated from endodontic infections and whether this feature can affect their adhesion to dentin. Therefore, this *in vitro* study sought to assess hydrophobicity levels in different root canal strains and clinical isolates derived from the lingual dorsum and their dentinal adhesion ability.

## Methodology

### *Candida albicans* isolates and growth conditions

A total of 32 clinical *C. albicans* isolates were tested for CSH. Sixteen of these strains were previously isolated from the lingual dorsum and 16 from necrotic root canals. The Research Ethics Committee of UFMG approved the protocol describing the specimen collection procedure of this study.

The species was identified using the standard methods of Yarrow, the taxonomic keys of Kurtzmann and Fell^22^ (1998), and the polymerase chain reaction (PCR) as previously described by Miranda, et al.^11^ (2009).

For the dentin adhesion assays, we randomly selected six *C. albicans* isolates from each niche that exhibited the lowest and highest hydrophobicity levels.

### Cell surface hydrophobicity assay

The CSH assay was performed using the microbial adhesion to hydrocarbon (MATH) test proposed by Rosenberg, Gutnick and Rosenberg^23^(1980) and adapted for yeasts. First, *C. albicans* isolates were grown in 0.34% yeast nitrogen base medium (Difco Laboratories, Detroit, MI, USA) supplemented with 250 mmol L^-1^ glucose for 24 h at 37 °C. After this period, one colony of each strain was resuspended in 2 mL KCl buffer (2 mmol L^-1^ KH_2_PO_4_, 5 mmol L^-1^ KCl, 1 mmol L^-1^ CaCl_2_, pH 6.8) and centrifuged twice at 5,000 rpm for 10 min at 4 °C. Yeast cells were then resuspended in the same buffer to yield an optical density of 0.3 (OD_before_) at 660 nm, which corresponds to 1x10^7^cells/mL^-1^.

**Figure 1:**
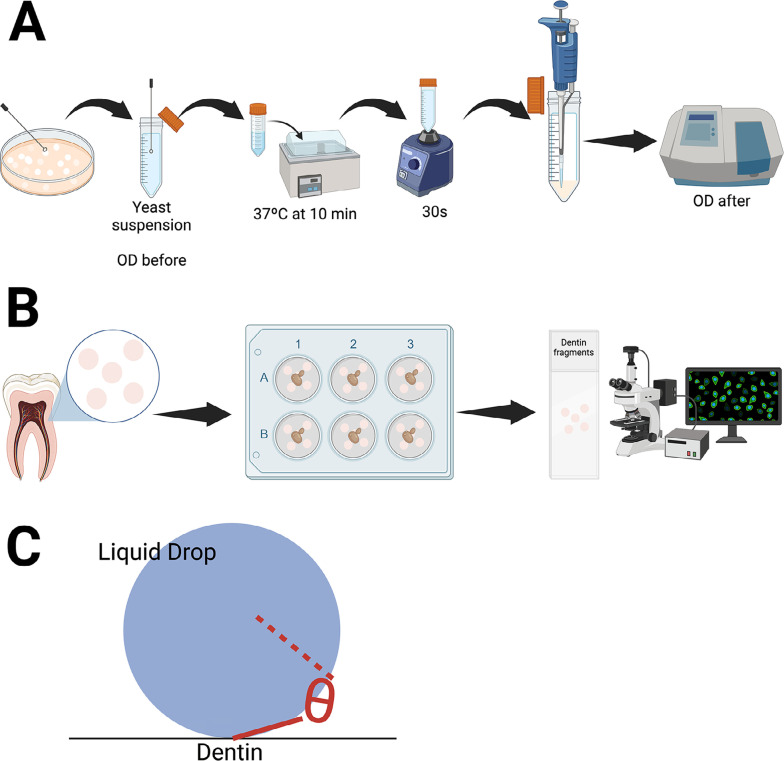
Mean hydrophobicity of *Candida albicans* strains isolated from the lingual dorsum and root canal. 1A: MATH assay for estimation of *C. albicans* hydrophobicity. 1B: An *ex vivo* assay to study *C. albicans* dentinal adhesion ability by using a fluorescence microscopy technique. 1C: The sessile drop method for measuring the hydrophobicity of the dentin surface.

For each strain tested, 2.5 mL of the suspension was added to two glass tubes (one test tube and one control tube). The microbial suspension was briefly overlaid with 0.5 mL of n-octane. The test and the control tubes were placed in a water bath at 37 °C for 10 min, removed, vortex mixed for 30 s, and returned to the water bath for an additional 30 min to allow separation of the immiscible n-octane and aqueous phase. The lower aqueous phase of the sample was carefully removed and transferred to a clean test tube (Figure 1A). Absorbance was measured at 660 nm (OD_after_) after vortex mixing and CSH was calculated using the formula:

Relative hydrophobicity $\text{ Relative hydrophobicity }(\%)=\left[1-\left(OD_{\text{before }}/OD_{\text{after }}\right)\right]\text{ x }100$

Suspensions without n-octane were used as negative controls. The assays were conducted on three separated occasions with duplicate measurements on each occasion.

### Dentin surfaces

We used non-carious third molars in this study. The surrounding enamel was removed with a high-speed industrial cutting instrument under copious water irrigation. Dentin discs (approximately 1.2 mm thick and 5 mm in diameter) were cut just near the occlusal-dentinoenamel junction using a rotary diamond saw (Isomet, Buehler, Lake Bluff, NY), providing discs that were ground on both sides (Figure 1B). Only one dentin disc was prepared per tooth. Complete removal of enamel was verified under a microscope (SZTP; Olympus Optical Co., Tokyo, Japan) at 15× magnification. The discs were ground with wet sandpaper (#400 to #1200 grit) to create smooth surfaces and to reduce the thickness of the discs to 1.0 mm measured with a micrometer (Miltex, Tuttlingen, Germany). This procedure created a smear layer on all surfaces of the discs. Standardization of each sample was confirmed by calculating the total surface area using the Image Pro-Plus 6.2 software (Media Cybernetics, Silver Spring, MD, USA).

### Dentin contact angle measurement

The sessile drop method was used to measure the hydrophobicity of the dentin surface. Contact angles were determined using a goniometer (Drop Shape Analysis System, DSA100, Kruess GmbH, Hamburg, Germany) equipped with a stainless-steel microsyringe needle. For static contact angle measurements, the microsyringe needle was positioned at 0.2 mm from the surface of the dentin sample. The volume of drop used was 1.5 μL and the distance between the needle deposit and standby position was 1.0 mm. After dispensing, the drop shape was monitored with a digital camera for 10 s at 18 frames per second and the contact angle was recorded. The goniometer measures the angle formed between the tangent line to the surface of the droplet at the contact point and the solid surface. (Figure 1C).

The measurements were performed at room temperature using three different liquids: water, formamide, and 1-bromonaphthalene. Each assay was performed in triplicate and at least 10 contact angles were measured per sample.

### Adhesion assay

The dentin discs prepared as indicated in item 2.3 were placed in a 24-well plate. Yeast cell suspensions were grown in Sabouraud dextrose broth (Sigma-Aldrich Corporation, San Luis, USA) overnight at 37 °C and the OD_600_ was adjusted to 1.0 (10^7^ cells/mL). Then, 2 mL of the suspension was added to each well. After 3 h of incubation in a shaker at 100 rpm and 37 °C, each well was washed twice with ultrapure water by carefully rinsing only the liquid over the disc. After the last wash, the liquid was completely removed. The discs were then stained with calcofluor white (Molecular Probes, Eugene, USA) for 5 min and observed under an epifluorescence microscope (Olympus IX 70, Tokyo, Japan). A total of 25 fields per sample were randomly captured with a video camera connected to the microscope and recorded by a computer. The Image Pro-Plus image analysis system was used to quantify the adhesion area (Figure 1B). Each experiment was repeated six times.

### Statistical analysis

Statistical analysis was performed using GraphPad Prism 8 (GraphPad Software Incorporation, San Diego, USA). Differences in relative CSH value and the dentin adhesion area between the *C. albicans* groups were evaluated using the Kruskal-Wallis test. Spearman’s rank correlation was used to test the correlation between CSH value and adhesion ability. Statistically significant differences were considered when p<0.05.

## Results

### Cell surface hydrophobicity of *C. albicans* isolates

**Figure 2:**
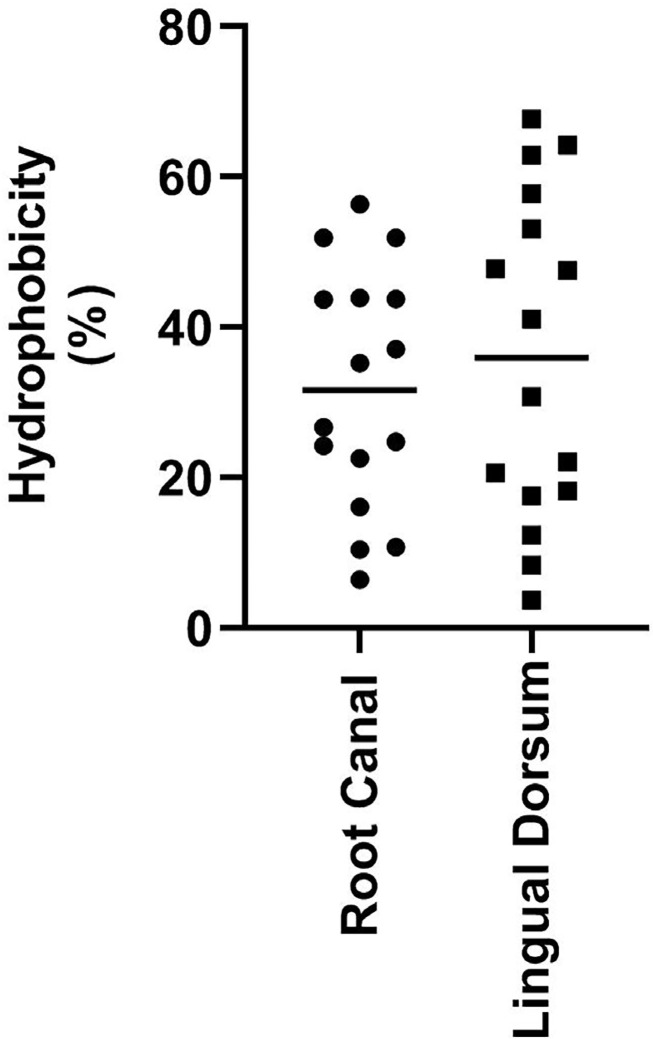
Mean area of adhesion of *Candida albicans* strains isolated from the lingual dorsum and root canal to the dentin substrate.

The CSH values of *C. albicans* isolates are presented in Figure 2. Regardless of the site of isolation, CSH was variable among the strains studied. Lingual dorsum isolates showed a mean relative CSH of 35.99%, ranging from 3.66 to 67.67%. The mean CSH of yeasts isolated from root canals was 31.61%, ranging from 6.44 to 56.33%. There was no significant difference in mean CSH values between isolation sites (p>0.05). Based on this hydrophobicity pattern, most isolates were characterized as moderately hydrophobic.

### Dentin adhesion

**Figure 3:**
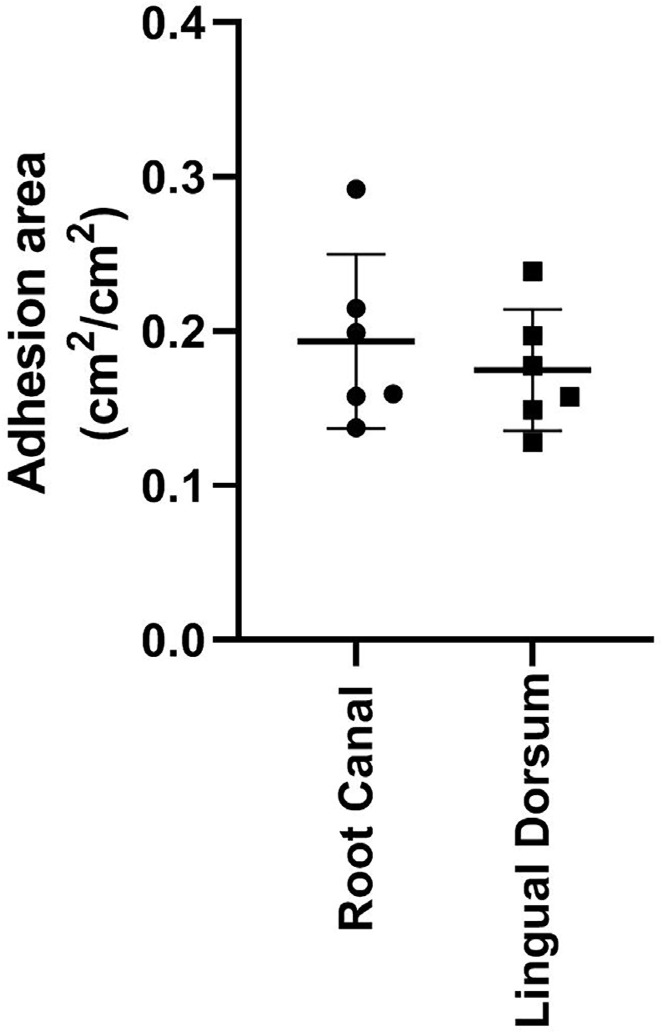
Spearman correlation analysis between hydrophobicity and dentin adhesion capacity of *Candida albicans* strains isolated from the lingual dorsum.

The adhesion areas of lingual dorsum isolates ranged from 0.1212 to 0.2518 cm^2^/cm^2^, while the areas of root canal isolates ranged from 0.1159 to 0.3533 cm^2^/cm^2^ (Figure 3). There was no significant difference in the ability to adhere to dentin between isolation sites (p>0.05).

**Figure 4:**
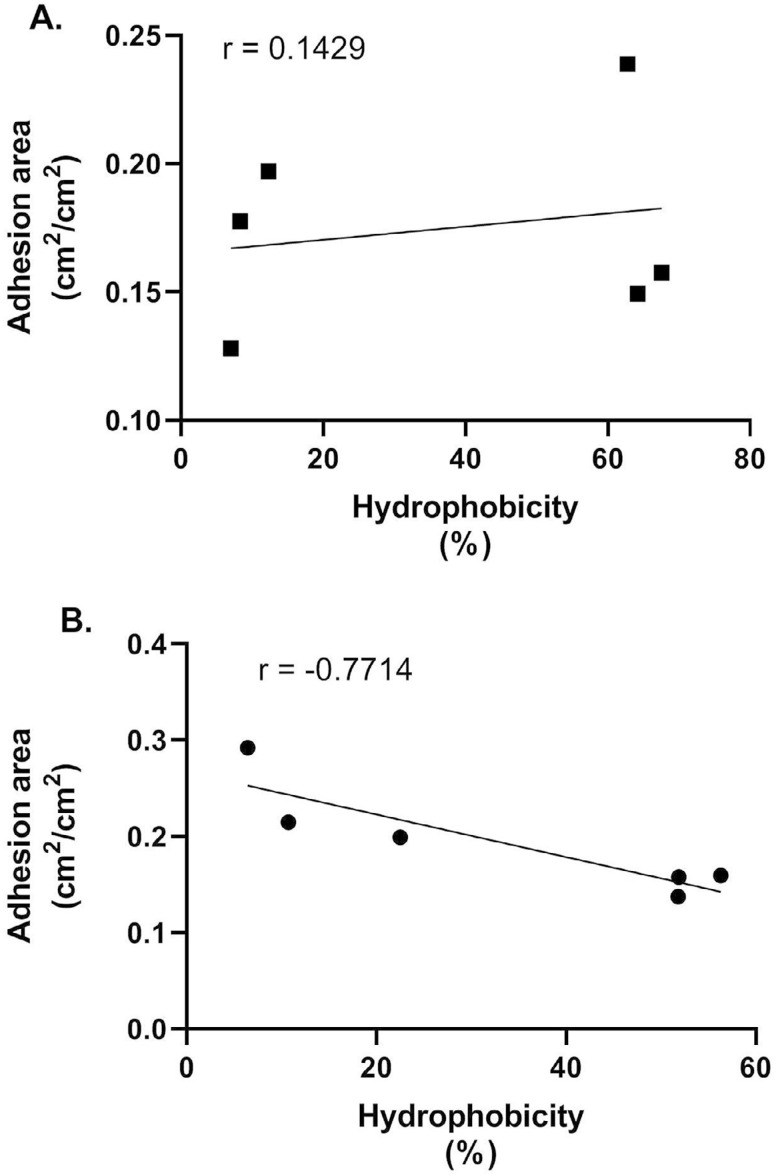
Spearman correlation analysis between hydrophobicity and dentin adhesion capacity of *Candida albicans* strains isolated from lingual dorsum(A) and root canal (B).

Correlation analysis of the results of adhesion to the dentin surface and relative CSH revealed a positive but nonsignificant correlation between these two parameters among lingual dorsum *C. albicans* (r=0.1429, p=0.8028), as indicated in Figure 4A. Among root canal isolates, there was a negative correlation between hydrophobicity and adhesion but it was not significant (r=-0.7714, p=0.1028) (Figure 4B).

**Figure 5:**
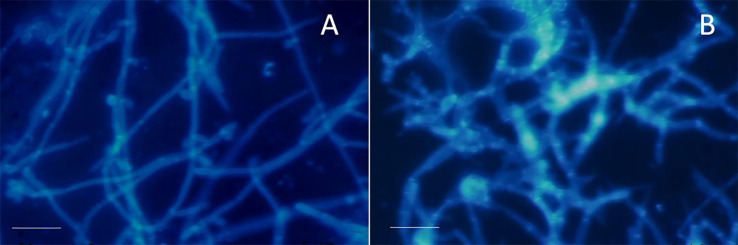
Micrographs of *Candida albicans* cells stained with calcofluor attached on dentin fragments with scale bars of 50 μm.

Figure 5 illustrates the adhesion of *C. albicans* to dentin fragments. The micrographs revealed that *C. albicans* isolates were preferentially adhered to dentin by hyphae morphology.

### Dentin surface hydrophobicity parameters

The contact angles formed by the three liquids (water, formamide, and 1-bromonaphthalene) on dentin surfaces are presented in Table 1. The values indicate a similar interaction of dentin with each of the three liquids tested. The water contact angle is an indicator of surface hydrophobicity. In this case, the water contact angles are lower than 50^o^ and the dentin surface can therefore be considered hydrophilic.

**Table 1:** Contact angles of water (θw), formamide (θf) and 1-bromonaphthalene (θb) on the dentin surface.

Substrate	Degree of contact angle		
	Water (θw)	Formamide (θf)	1-Bromonaphthalene (θb)
**Dentin**	39°±4°	42°±3°	45°±3°

## Discussion

For many decades, microbiologists have focused their attention on the involvement of bacteria in endodontic diseases. However, in recent years, there has been increasing interest in fungal microorganisms. It is evident that fungi contribute to a subset of root canal infections,^4,10,11^ although the existing body of evidence remains relatively modest. The prerequisite for the onset of dentinal tubule invasion by *C. albicans* is the adhesion of the yeast to dentin.^14^ This adhesion process involves various non-specific factors, including attractive and repulsive forces such as van der Waals forces, hydrophobic interactions, and Brownian movement forces. Additionally, electrostatic interactions play a pivotal role in facilitating initial adherence.^24^ In this study, we sought to quantify cellular hydrophobicity to establish the relationship between the adhesion capacity of oral *C. albicans* isolates and the characteristics of their cell surfaces. We selected the MATH method as originally proposed by Rosenberg, Gutnick and Rosenberg^23^(1980) for this purpose because of its practicality and ease of use within the scope of our research.

Previous studies have proposed numerical correlations between the percentage of cells adhering to hydrocarbon and the hydrophobicity levels of microorganisms.^25,26^ Within this context, it was established that microbial strains with high hydrophobicity show adhesion percentages equal to or exceeding 50%. Samples with readings between 20% and 50% are characterized as moderately hydrophobic, while cell surfaces with hydrophilic traits show percentages of 20% or less. By employing these benchmarks, our results reveal that most *C. albicans* strains isolated from both the lingual dorsum and the necrotic root canal are characterized by moderate hydrophobicity. According to Hazen and Hazen^27^ (1988), the incubation temperature applied in our study facilitated the observation of hydrophobic behavior of the microorganisms. Souza, et al.^28^ (2009) suggested that the modulation of *C. albicans* hydrophobicity by temperature may lead to misconceptions regarding this parameter. However, most CSH studies^29,30^recommend a temperature of 37 °C, as employed in our study, to be the most suitable for evaluating hydrophobicity in dimorphic fungi.

As highlighted by Lai, et al.^31^ (2022), individual variations in the microbial growth curve might have influenced the hydrophobicity values obtained in the present study. After 24 h of incubation, most *C. albicans* strains tested were in the exponential phase of microbial growth. At this stage, the protein-mannosylated cell wall fibrils increase in length and concentration, promoting the expression of hydrophobic behavior. In contrast, yeasts in the stationary phase show a hydrophilic profile.

The increased formation of germ tubes, pseudo-hyphae, and hyphae by oral yeasts, facilitated by the nitrogen-containing culture medium, may also be associated with the inherent hydrophobicity of most strains investigated. Studies^31-34^reported variation in the hydrophobic profile of *C. albicans* yeasts according to cellular morphology, with pseudo-hyphae exhibiting variable hydrophobicity, while true hyphae manifest high hydrophobicity. Since pleomorphism is inherently associated with the colonization capacity of *C. albicans* in the dentin environment, we expected that root strains would display hydrophobic behavior, as observed for most yeasts isolated from this niche.^15-18^ The findings of Hazen and Hazen^27^ (1988) indicate a direct correlation between hydrophobicity and the pathogenic potential of the sample suggest a significant role for *C. albicans*, with a high adhesion percentage to n-octane, isolated from root canals, in the initiation and progression of primary apical periodontitis. These authors also highlight that the transient expression of hydrophobic behavior under specific environmental conditions may enable yeasts within the endogenous microbiota to colonize alternative sites, initiating an infectious process. This implies that strains isolated from the lingual dorsum, whose adhesion percentage to hydrocarbons exceeds 20%, possess the potential for pulp colonization.

In this study, we also assessed the water affinity characteristics of dentin using the contact angle method. Liber-Kneć and Łagan^35^ (2021) suggested contact angle measurement to be the most suitable approach for evaluating the hydrophobicity of substrates in microbial adhesion assays. Following previously established parameters, the results indicated that dentin, with a contact angle of 39°, showed a hydrophilic behavior. Similar results were reported by Henriques, Azeredo and Oliveira^36^ (2004) for substrates such as hydroxyapatite and acrylic. Differences in contact angle values are due to variations in surface topography, the surface tension of the liquid used in the experiments, substrate surface energy, and the level of interaction between the liquid and the solid.^37^

Since CSH can affect both the adhesion and the pathogenic processes of *C. albicans*,^19,30,38^ adhesion assays were conducted using *C. albicans* strains obtained from the lingual dorsum and the root canal, which exhibited hydrophilic behavior (adhesion percentage to n-octane < 20%) and high hydrophobicity (adhesion percentage to n-octane > 50%), respectively. To the best of our knowledge, there is no report evaluating the CSH of root canal *C. albicans* isolates and their dentin adhesion ability.

Regardless of the primary isolation site, both hydrophilic and hydrophobic isolates were able to adhere to dentin. However, contrary to the findings of Panagoda, Ellepola and Samaranayake^39^ (1998), we observed no correlation between cellular hydrophobicity and microbial adhesion capacity. Raut, Rathod and Karuppayil^18^ (2010) also did not find any correlation between these parameters.

In our study, the effective adhesion of yeast to dentin (even without the addition of saliva and within a short incubation period) suggests that saliva may play a potential enhancing role but is not determinant for adhesion, as also reported by Henriques, Azeredo and Oliveira^36^ (2004). Saliva contributes to reducing the electrostatic repulsion force between the yeast and the substrate by absorbing mucins, secretory IgA, and other proteins. Nevertheless, Gunaratnam, et al.^40^ (2021) found that all adhesion parameters were enhanced on the salivary pellicle-covered compared to the uncovered enamel.

Our study also showed intraspecific variations in the adhesion capacity of *C. albicans* strains.^40,41^ According to Suchodolski, et al.^26^ (2020), genes encoding adhesion molecules are not uniformly expressed across all *C. albicans* strains. This gene expression is individually influenced by changes in pH, carbon, and nitrogen supply.

Although this study was not focused on examining bacterial attachment, it is important to mention that in clinical settings, *C. albicans* commonly coexists with bacteria in dental caries and in the root canals infections. Some investigations^42,43^indicate an increased prevalence of *S. mutans* in oral biofilms at sites where *C. albicans* is also present. The biofilm matrix is thereby identified as a key factor for co-aggregation between *S. mutans* and *C. albicans*. Insoluble and soluble glucans are the main components of extracellular polysaccharides (EPS) and are essential for forming the core of the biofilm matrix.^43^ Furthermore, *S. mutans* glucosyltransferases exoenzymes expression is induced by the presence of *C. albicans* in mixed-species biofilms. The matrix facilitates accumulation and adherence to the tooth surface, thus increasing the virulence of *S. mutans* and *C. albicans* for its host.^42^

Interestingly, *E. faecalis* has been shown to incorporate itself into *C. albicans* biofilms, adhering to both yeast and hyphal forms. Bacteria preferentially adhere to hyphal as opposed to yeast cells. This interaction may favor resistance to treatment through enhancing dentinal tubule penetration. At molecular levels, *E. faecalis* downregulated key genes involved in *C. albicans* virulence, whilst *C. albicans* upregulated genes involved in *E. faecalis* adhesion and biofilm formation.^44^

Despite differences in hydrophobicity levels, in our study, all *C. albicans* isolates demonstrated some adhesion capacity. However, the behavior of these microorganisms in dentin should be further investigated, particularly using *in vivo* experiments. Under these conditions and in the presence of other microbial groups and oral fluids, *C. albicans* may show distinct morphological responses, with variable implications for the pathogenesis of endodontic and periradicular infections.

## Conclusion

Hydrophobicity is not a pivotal factor influencing the adhesion of oral *Candida albicans* strains to dentin. Further studies are necessary to better understand the mechanisms underlying the adhesion of *C. albicans* to dentin.
